# Stigmatisation of people experiencing gambling-related harms: a vignette study of the predictors of desire for social distance

**DOI:** 10.3389/fpsyg.2025.1613798

**Published:** 2025-06-18

**Authors:** Joanne Lloyd, Katy L. Penfold, Darren David Chadwick, Laura Louise Nicklin, Daniel P. Hinton, Sokratis Dinos

**Affiliations:** ^1^School of Education and Psychology, University of Wolverhampton, Wolverhampton, United Kingdom; ^2^School of Psychology, Liverpool John Moores University, Liverpool, United Kingdom; ^3^National Centre for Social Research (NatCen), London, United Kingdom

**Keywords:** gambling, stigma, vignette, addiction, gambling harm

## Abstract

**Introduction:**

Stigma is associated with psychological distress and can act as a barrier to help-seeking for people who experience gambling harms. While research into intersectional stigma within this population is scarce, this may be exacerbated for those from multiply-marginalised groups.

**Method:**

This study used an online survey with ‘vignette’ design to capture attitudes of 3,567 adults in Great Britain towards hypothetical individuals experiencing gambling harms alongside a variety of other potentially stigmatised characteristics (minority ethnicity, LGBTQ status; low-income status; chronic drug/alcohol use; and mental health difficulties). Questions about participants’ own demographic characteristics, their contact with and experience of gambling/gambling harms, and their beliefs about the nature and origin of gambling harms were also administered.

**Results:**

Significantly greater desire for social distance from protagonists experiencing gambling harms than those described as gambling recreationally (*p* < 0.05) indicated the presence of public stigma, and this was further elevated (*p* < 0.05) when the protagonist was described as having difficulties with drug and alcohol use. The other potentially stigmatised characteristics were not associated with an additional increase in stigma, and potential reasons for this are discussed. Perceived disruptiveness and harmfulness of the protagonist, along with beliefs that gambling harms are due to bad character and difficult to recover from, were all significant predictors of desire for social distance – as were lower levels of prior contact with gambling harms (all *p* < 0.05).

**Discussion:**

These findings have utility for stigma reduction interventions, and can also inform those working with people experiencing gambling harms.

## Introduction

Around 44% of the adult population of the UK participate in gambling (Muggleton et al., 2021; Gambling Commission, 2023), and while some do not experience any harms, others experience symptoms of ‘problem gambling’ and/or a variety of gambling-related harms (Gabellini et al., 2022) which can be severe and enduring (Rockloff et al., 2022). Gambling harm is an important public health issue (Johnstone and Regan, 2020; Bowden-Jones et al., 2022), and while reasonably efficacious interventions exist (Eriksen et al., 2023) only a small proportion of people experiencing gambling harm seek treatment (Bijker et al., 2022), and often only after several years of harms (Grant and Chamberlain, 2024), or once harms are severe (Evans and Delfabbro, 2005; Bijker et al., 2022).

Barriers to treatment-seeking for gambling harms are complex and variable (De Vos et al., 2021), but stigma, defined as ‘*labelling, stereotyping, separation, status loss, and discrimination in a context in which power is exercised’* (Donaldson et al., 2015, p.165) has consistently been identified as a major barrier (Evans and Delfabbro, 2005; Suurvali et al., 2009; Gainsbury et al., 2014; Leslie and McGrath, 2024). While gambling is a popular, legitimised and normalised leisure activity within Great Britain (Orford, 2018; McGee, 2020), those who experience harms from gambling are subject to stigma (Quigley, 2022). Education and prevention campaigns for gambling harm typically align with ‘responsible gambling’ discourses, where the onus for gambling ‘safely’ is placed upon the individual, with those who fail to do so positioned as blameworthy, and as a disordered minority (van Schalkwyk et al., 2022; Wyllie et al., 2023).

Stigma is associated with psychological distress and has been conceptualized as a form of gambling harm in and of itself (Langham et al., 2015), and research on other stigmatised conditions has demonstrated an inverse relationship with quality of life (e.g., Degnan et al., 2021). Stigma is a multi-faceted construct (Link, 2001), and several kinds of stigmatisation [which often co-occur; (e.g., Hing and Russell, 2017a)] are encountered by people experiencing gambling harms, including *experienced* (or ‘*enacted’*) stigma (referring to having been treated in a negative or discriminatory way due to stigmatisation); *perceived* or *anticipated* stigma (referring to people’s perceptions of the stigmatising beliefs the general public holds); and ‘*self-stigma*’ or ‘*internalized stigma*’ (referring to internalized negative attitudes about oneself) (Donaldson et al., 2015).

Goffman’s seminal work identified labelling, stereotyping, separation, status loss, and discrimination as the key processes underpinning stigma (Goffman, 1963). Via labelling and stereotyping, groups or individuals who possess certain attributes or identities are viewed as deviant, inferior, or ‘spoiled’, which leads to a desire for ‘separation’ (Goffman, 1963). Later work has used the term ‘desire for social distance’ to capture this concept of unwillingness to engage socially that is a core component of stigma, and highlighted how particular kinds of stereotypes, including ‘dangerousness’ are key in driving desire for social distance from the stereotyped group (Corrigan et al., 2003).

Aranda et al. (2023) emphasise the relational nature of stigma, i.e., the fact that it is not simply localised within the stigmatised group; but exists within the interactions and relationships between ‘audiences’ and ‘targets’ [‘in the nexus of social interactions among divers e social audiences’ (Aranda et al., 2023, p.1339)]. To comprehensively understand stigma, therefore, we must consider the people and mechanisms that create or perpetuate stigma, in addition to the experiences and characteristics of those who encounter it. Corrigan outlined how causal attributions, where people are viewed as being ‘to blame’ for the stigmatised condition, can generate anger and a desire to punish the individual through stigmatising treatment; as well as how perceived dangerousness or harmfulness of an individual or group generate fear and a desire for distance in order to avoid perceived risk of harm, (Corrigan et al., 2003).

To measure individuals’ stigmatising attitudes towards others, vignettes are typically used to describe a hypothetical individual, followed by questions to evaluate beliefs and attitudes related to stigma; primarily via reported desire for social distance (Fox et al., 2018a), sometimes alongside questions to prompt about beliefs about the nature and severity of the condition described in the vignette (Hing et al., 2016b).

Using such methods, specific beliefs about stigmatised populations have been found to predict stigmatisation, including within the gambling literature: Believing gambling harms to be disruptive, noticeable, and/or difficult to recover from; believing that people experiencing gambling harms might harm someone; and believing gambling harms to be due to bad character are all associated with greater levels of stigmatisation in the form of greater desire for social distance (Hing et al., 2016a).

In terms of characteristics of the person making judgements about a stigmatised group, level of familiarity or prior contact with someone experiencing gambling harms has been found to be inversely associated with stigma (Dhillon et al., 2011) – mirroring findings from the wider literature on intergroup relations (White et al., 2021), although the effect is relatively small (Hing et al., 2016a), and not all studies have found it to be significant (Delfabbro et al., 2021b) – possibly because it has the potential to have both positive and negative impacts on stigmatising attitudes (Delfabbro et al., 2021a), e.g., due to associative stigma and burden at high levels of familiarity – sometimes resulting in a ‘U’ shaped relationship between familiarity and stigma (Corrigan and Nieweglowski, 2019). There has been relatively little research into the stigmatisation of others experiencing gambling harm by those with lived experience of harm themselves, with most research with this population focusing on experiences of stigmatisation and/or perceptions of stigmatisation by the general population (Dąbrowska and Wieczorek, 2020). Extrapolating from findings that level of prior contact predicts reduced stigma, and from one study that found gambling experience to be associated with lower desire for social distance (Hing et al., 2016a), we might expect less stigmatisation of others by those with lived experience of harms, but findings of high levels of self-stigma and perceived stigma in this population (Dąbrowska and Wieczorek, 2020) along with the phenomenon of ‘self-group distancing’ which has been identified in the wider literature on stigmatised social groups (van Veelen et al., 2020) could also suggest that this would translate to greater desire for social distance from others experiencing gambling harms.

In terms of the stigmatised individual, certain demographic and other characteristics may increase the degree of stigmatisation (Fox et al., 2018b), and in recent years there has been growing recognition of the ‘need to recognise an individual’s membership in multiple stigmatised groups’ (Turan et al., 2019, p. 2) in order to better understand intersectional stigma (Oexle and Corrigan, 2018). Age, socioeconomic status, gender, sexuality, and ethnicity, for instance, while not an exhaustive list, may all be relevant variables for consideration, alongside mental health conditions and alcohol or substance use.

Few studies have explored age in relation to gambling-related stigma (Wöhr and Wuketich, 2021), but a few have found older age to be related to more stigmatising views about people experiencing gambling harms (Hing et al., 2016a; Rockloff and Schofield, 2004) and another found a positive correlation between age and self-stigma (Hing and Russell, 2017b). The same study did not find education nor income to be significant predictors of stigma (Hing and Russell, 2017b). However, there are established links between financial deprivation and gambling harms (Moss et al., 2023; Saunders et al., 2023) and those who experience gambling harms alongside poverty – particularly homelessness, can be heavily burdened by stigma (Hahmann et al., 2021). Furthermore, some treatment providers described increased stigmatisation of those with a lower perceived ‘social status’ (Dąbrowska and Wieczorek, 2020).

Evidence for a relationship between gender and stigma is mixed; some studies have found no significant gender differences in perceived stigma (Andrà et al., 2022) or self-stigma (Gavriel-Fried and Rabayov, 2017), whereas some have found that females experience greater self-stigma related to gambling harms (Hing et al., 2015; Hing and Russell, 2017b). Women with children may be at particular risk of stigma, (Fannin et al., 2024) due to perceptions that people experiencing addiction and/or gambling harms are ‘socially undesirable’ or ‘negligent’ mothers (Collinson and Hall, 2021; Estévez et al., 2023). There is little explicit research into stigmatisation of gambling harms amongst people from a sexually- or gender- diverse population, but the wider research indicates that these groups are more likely to experience stigma and related stress in general (Diamond and Alley, 2022), and stigma related to sexual and gender identity is a risk factor for gambling harms (Bush et al., 2021).

While gambling participation is lower amongst minority ethnic groups in the UK, a greater proportion of those who do gamble experience harms – potentially driven partly by experiences of racism and discrimination, and use of gambling as a coping strategy (Moss et al., 2023). Racism and discrimination are more prevalent amongst those who gamble and/or experience gambling harms [those scoring 1 + on the Problem Gambling Severity Index (PGSI; Ferris and Wynne, 2001)] than those who do not, suggesting people from minority ethnic groups may be at increased risk of stigmatisation of gambling harms.

Gambling harms are also known to be frequently comorbid with substance and alcohol use and also with mental health conditions (Butler et al., 2020; Wardle et al., 2020; Roberts et al., 2021). Substance and alcohol use and mental health conditions are both, individually, associated with stigma (Volkow et al., 2021). This highlights the potential for compounded/intersectional stigma amongst those experiencing multiple difficulties – although, again, there is little extant literature directly exploring gambling-related harms stigma associated with these intersectional characteristics.

The current study used a ‘vignette’ design to capture attitudes of a sample of the general population of Great Britain, towards hypothetical individuals experiencing gambling harms alongside a variety of other potentially stigmatised characteristics (namely; minority ethnicity, LGBTQ status; low-income status; chronic substance/alcohol use; and mental health difficulties). By measuring participants’ desire for social distance from individuals depicted in the vignettes, along with their beliefs about the nature and origin of gambling harms, and their own demographic characteristics and level of prior contact with and experience of gambling harms, we aimed to (a) estimate the nature and extent of stigmatisation of people who experience gambling harm, and (b) identify predictors of desire for social distance, in terms of characteristics of the hypothetical stigmatised individual, as well as characteristics of the participant. We predicted that a protagonist experiencing gambling harms would attract greater desire for social distance than a protagonist in a control condition (a person gambling without harms), and that information about all additional stigmatised socio-demographic characteristics would further increase the desire for social distance. We tentatively predicted that there would be greater stigmatisation of female compared with male protagonists, and that prior contact with gambling harms, and experience of gambling harms, would be associated with less desire for social distance.

## Materials and methods

### Design

The study design was a cross-sectional survey, with a between-subjects experimental vignette component (where participants read one of 14 possible versions of a vignette, about which they then answered a series of questions).

### Materials

#### Demographic items

Demographic information was collected using the question wordings and response options described in the Annual GB Treatment and Support Survey (Gunstone et al., 2021). Participants’ age, sex, and ethnicity; alongside a variety of other demographic characteristics not reported here, were taken from their YouGov panel information.

#### Gambling-related items

Participants were asked whether they had gambled (on a comprehensive list of activities, not reported here), over the past 12 months. Those who had gambled on one or more activity were administered the widely-used 9-item PGSI (Ferris and Wynne, 2001), which is used to measure symptoms of ‘problem gambling’ and has a Cronbach’s alpha of >0.8 (e.g., Tseng et al., 2023). Response options were: ‘Never’, ‘Sometimes’, ‘Most of the time’, and ‘Almost Always’, with a possible score from 0 to 27. Those who had not gambled in the past 12 months were given a score of 0.

Participants were also asked ‘do you think anyone you know has, or previously had, a problem with their gambling? This could include family members, friends, work colleagues or other people you know’. Those who responded ‘yes’ were then asked ‘do you feel you have personally been negatively affected in any way by this person/these people’s gambling behaviour? This could include financial, emotional or practical impacts.’ Those who also responded ‘yes’ to this question were classified as an ‘affected other’ for the purposes of the analyses that follow.

#### Vignettes and associated measures

Vignettes described a male or female protagonist in seven different scenarios (see Table 1 for summary of conditions and wordings), i.e., there were 14 possible vignettes, and each participant was randomly allocated one. The main text of the vignettes was based on those used by Hing et al. (2016b), with vignettes 1 and 2 describing someone gambling without experiencing harms, vignettes 3–4 describing someone experiencing symptoms of ‘gambling disorder’, and vignettes 5–14 describing someone experiencing ‘gambling disorder’ alongside one of 5 additional characteristics. The ‘gambling disorder’ symptoms were based on criteria from the DSM-5 (American Psychiatric Association, 2013). These characteristics were described as concisely as possible, in order to maintain the primary focus on the gambling information, and keep each vignette of a comparable, concise length. Initials (‘AJ’) were used rather than a forename for the protagonist, to avoid biasing participants’ assumptions about age, social class or ethnicity.

**Table 1:** Summary of vignette conditions.

Vignette conditions		Wording
(1) Gambling, no harms, male	(2) Gambling, no harms, female	AJ is a [wo]man who lives in your community, who enjoys watching movies, reading, and spending time with friends and family. During the last year, AJ has started to gamble occasionally. [S]he usually bets the same amount of money and never bets more than [s]he intends. [S]he stops gambling when [s]he is losing and does not lose very much money. [S]he often goes long periods without gambling and does other leisure activities instead. [S]he does not find [s]he misses gambling and [s]he does not think about gambling while [s]he is away from it. AJ’s family and friends know that [s]he sometimes gambles.
(3) Gambling harms, male	(4) Gambling harms, female	AJ is a [wo]man who lives in your community, who enjoys watching movies, reading, and spending time with friends and family. During the last 12 months, [s]he has started to gamble more than his/her usual amount of money. [S]he has even noticed that [s]he needs to gamble much more than he used to in order to get the same feeling of excitement. Several times, [s]he has tried to cut down, or stop gambling, but [s]he cannot. Each time [s]he has tried to cut down, [s]he became agitated and could not sleep, so [s]he gambled again. [S]he is often preoccupied by thoughts of gambling and gambles more to try to recover his/her losses. AJ has also hidden the extent of his/her gambling from his/her family and friends.
(5) Gambling harms + identifies as LGBTQ, male	(6) Gambling harms + identifies as LGBTQ, female	As vignettes 3 & 4, with the addition of “AJ identifies as LGBTQ” inserted after the first sentence.
(7) Gambling harms + mental health problems, male	(8) Gambling harms + mental health problems, female	As vignettes 3 & 4, with “AJ was hospitalised due to mental health problems (including depression and anxiety) about 6 months ago” inserted after the first sentence.
(9) Gambling harms + drug and alcohol use, male	(10) Gambling harms + drug and alcohol use, female	As vignettes 3 & 4, with “AJ has been using heroin and consuming large amounts of alcohol for the past 8 years” inserted after the first sentence.
(11) Gambling harms + minority ethnicity, male	(12) Gambling harms + minority ethnicity, female	As vignettes 3 & 4, with “AJ identifies as a member of an ethnic minority group in the UK” inserted after the first sentence.
(13) Gambling harms + low income, male	(14) Gambling harms + low income, female	As vignettes 3 & 4, with “AJ is from a low-income household” inserted after the first sentence.

After being presented with one of the vignettes, participants received a series of questions about their attitudes towards the hypothetical individual described - including a validated measure of social distance, and items that have been used to measure constructs associated with, or predictive of, stigma in other studies (i.e., perceptions of harmfulness, noticeability, recoverability and disruptiveness, and beliefs about origins of harm): The specific measures used were as follows.

#### The social distance scale (SDS)

The social distance scale (Martin et al., 2000) is a 6-item measure of desire for social distance (with a Cronbach’s alpha of >0.85 when applied to gambling (Hing et al., 2016a)), where respondents rate how willing they would be to do things like ‘be friends with’ or ‘live next door to’ the individual described (e.g., in a vignette). Each item is scored from 1 (definitely willing) to 4 (definitely unwilling), giving a total score out of 24, with higher scores indicating greater desire for social distance from the hypothetical person (− a proxy measure for stigmatisation and discrimination). Cronbach’s alpha for this scale in the current study was 0.89.

#### Perceived harmfulness

Two items probed about perceived harmfulness of the individual described (based loosely on Horch and Hodgins’ (2008) ‘perceived dangerousness’ item (Horch and Hodgins, 2008), adjusted in consultation with our lived experience panel to better apply to gambling). The first asked ‘How likely do you think it is that X would cause hurt or harm to other people?’ and the second ‘How likely do you think it is that X would cause hurt or harm to themselves’. They were scored from 1 (extremely unlikely) to 5 (extremely likely), with higher scores indicating greater perceived harmfulness of the individual in the vignette.

#### Perceived noticeability

A single item taken from Hing et al. (2016) probed about the perceived noticeability/concealability of the individual’s situation (Hing et al., 2016b). Responses were scored from 1 (not at all noticeable) to 5 (extremely noticeable), i.e., higher scores indicated a belief that the situation was more noticeable. Due to the wording of this item (‘How noticeable would X’s situation be to their family and friends if they had not told them about it?’) this was only presented after vignettes 3–14 (i.e., not after the vignette describing a person not experiencing harm).

#### Perceived disruptiveness

A 4-part item was adapted from the ‘Key Informants’ Questionnaire’ (Alem et al., 1999) to apply to gambling (Hing et al., 2016b), to assess perceived disruptiveness of the situation, which asks participants to rate how much they think the individual’s situation would impact their ability to live independently, be in a serious relationship, work/study, or be successful. The last option (‘be successful’) was added, in response to lived experience panel input. Responses were scored from 1 (not at all) to 4 (a large amount), with higher scores indicating higher perceived disruptiveness. Again, this was only presented to those receiving vignettes 3–14 (as it did not make sense to ask about the individual described in the ‘no harms’ vignette conditions).

#### Perceived causes

A 6-part question asked participants (who received vignette 3–14) to rate the extent to which they attribute the hypothetical individual’s circumstances to each of six different causes (‘bad character’; ‘chemical imbalance in the brain’; ‘stressful life circumstances’; ‘genetic/inherited problem’; ‘god’s will’; and ‘the way they were raised’). This was based on the ‘Perceived Causes’ scale (Link et al., 1999) used in relation to gambling by Hing and colleagues (Hing et al., 2016b). Responses were scored from 1 (extremely unlikely) to 5 (extremely likely), i.e., a higher score on a given item indicated greater belief in that cause having been the reason for the gambling harms.

#### Perceived recoverability

A single item (again only presented to those receiving vignettes 3–14) probed about the perceived recoverability of the individual from their situation (Hing et al., 2016b). Responses to the question ‘How strongly do you agree or disagree that people can recover from AJ’s situation?’ were scored from 1 (‘strongly disagree’) to 5 (‘strongly agree’), i.e., higher scores indicated a greater belief that the hypothetical individual could recover from their situation.

#### Level of prior contact measure

This was adapted from Holmes et al. (1999) ‘level of contact report’. In order to measure the degree of familiarity/contact participants had with people experiencing gambling harms, they were asked to tick off which of 12 types of contact they have had (options include things like having had contact with a colleague/relative/member of the household experiencing gambling harms – indicating greater degree of contact, and having seen a documentary about someone experiencing gambling harms – indicating a more distant level of contact). We modified language slightly to make it more accessible and current, and in response to lived experience panel and expert advisors’ recommendations. Specifically, we merged two items which referred to having experience of working in service provision for people with gambling harms, as stakeholders found it difficult to see the very subtle difference between them. We also modified item 3 (which refers to having seen a character experiencing gambling harms on TV or in movies) to include ‘in books’, and item 4 (which refers to having seen a real person experiencing gambling harms in a documentary or article) to include ‘on social media’. Scoring was hierarchical, ranging from 1 (no experience of contact at all) to 11 (first-hand lived experience). In line with Holmes, participants received the score corresponding to the greatest degree of contact that they reported (regardless of how many other forms of contact they reported), so scores ranged from 1 to 11.

### Procedure

Participants were recruited from YouGov’s existing panel of 400,000 survey-takers, who had consented to receiving study invitations. YouGov emailed potential participants a link to the full information sheet. If they choose to take part, they provided online consent and proceeded to the survey. They were then presented with all relevant scales detailed above (see individual scale descriptions for which measures were administered to whom; some applied to all participants, and some only to those who had gambled in the past 12 months, for example). At the end of the survey, participants were presented with a debrief and signposted to relevant support services. Participants received ‘points’ for taking part (at a tariff explained in their existing agreements with YouGov) which can be accumulated and redeemed for cash.

### Ethics statement

Ethical approval was received from the Psychology Ethics Panel at the University of Wolverhampton (REF: 0523JLUOWPSY) and from NatCen’s Research Ethics Review Committee (REF P17783). Online confirmation of informed consent was required before participants proceeded to the survey.

### Analysis

#### Data and weighting overview

YouGov supplied a dataset of *n* = 3,567 participants, created by merging two cleaned and weighted datasets from their August 2023 Treatment and Support Survey conducted for GambleAware. These were a weighted general population sample (*n* = 3,276), and a further ‘boost’ sample of people who experience gambling harm (*n* = 796) (with PGSI scores of 1+). The two samples were combined to maximise sample size for the current study, and as such the weightings were no longer used as they were not applicable to the merged sample, which is thus not strictly representative of the GB population.

#### Analysis

A series of 2-way ANOVAs were conducted to test for main effects of gender and other vignette characteristics on the aforementioned variables associated with stigma; i.e. desire for social distance, and beliefs about origin, recoverability, harmfulness, disruptiveness and noticeability. Due to violation of parametric assumptions for some variables, data were analysed using robust two-way independent factorial ANOVAs based on a 20% trimmed mean, using the WRS2 package in R (Mair and Wilcox, 2020). We used trimmed means as there were a large number of outliers and heavy skew for some of the variables. In these circumstances, trimmed means are preferable to Winsorised means or M-estimators. Where main effects were significant, post-hoc multiple comparisons to identify which vignette conditions differed from one another were conducted using the Games-Howell test in SPSS – this t-test does not rely on equal variances and sample sizes, as it uses ranked variables. It is based on Welch’s degrees of freedom and controls for type I error for the entire comparison and is considered particularly appropriate where 6 or more conditions are compared with >50 cases per group (Lee and Lee, 2018) – i.e. the conditions present in this analysis. Crosstabulations were used to explore differences in the proportion of participants endorsing particular beliefs/attitudes, based on various factors (e.g., to compare proportion of people with and without lived experience of gambling harms who would be willing to engage with someone experiencing gambling harms), with significance tests (Chi-squared/Fisher’s Exact Test) where relevant.

In order to explore how multiple factors combined to influence desire for social distance, a hierarchical linear regression with ‘desire for social distance’ as the criterion variable was also conducted, with appropriate assumption checks. Following a similar method to Hing et al. (2016a), block 1 contained scores on stigma-related constructs (beliefs about origin, recoverability, disruptiveness, harmfulness and noticeability), and block 2 contained information about demographic characteristics and prior contact with gambling harms (with binary variables representing lived experience and experience an affected other, and a continuous variable for level of prior contact).

## Results

### Descriptive analysis of sample characteristics

The sample consisted of 1,698 (47.6%) males and 1869 (52.4%) females, with a mean age of 47 (range 18–88, SD 17.34). Ethnicity of the sample was broadly representative of the GB population, though White British participants (88.1%) were somewhat over-represented compared with the UK 2021 Census figure of 81.7%, and other groups were, conversely, under-represented, with ethnicity of 5.7% of our sample being Asian; 2.6% being Black; 3.4% Mixed and 0.3% Other ethnic groups. A total of 2,400 participants had gambled in the past 12 months and 1,167 had not. Of those who had gambled, *n* = 1,604 had a PGSI score of 0; *n* = 301 scored 1–2; *n* = 262 scored 3–7; and *n* = 262 scored 8+. A total of 945 people reported that they knew (or had known) someone who ‘had a problem with their gambling’, and 324 reported being affected by that person’s gambling. Of these ‘affected others’, 240 had gambled in the past 12 months and 84 had not gambled.

### Desire for social distance across different vignette scenarios

Desire for social distance from the hypothetical individuals in the vignettes ranged from 6–24, with higher scores representing greater desire for distance (a proxy for greater stigma). Robust 2-way ANOVA indicated that there was a significant main effect of vignette (*Q* = 593.47, *p* = 0.001) but no significant main effect of gender (*Q* = 3.35, *p* = 0.068) and no significant interaction between gender and vignette (*Q* = 3.43, *p* = 0.76). Table 2 summarises the mean desire for social distance across vignettes, with data from the male and female vignettes combined, given the absence of vignette gender effects (all *p*-values were >0.05). It also presents a breakdown of proportion of people who did not desire social distance in each scenario (i.e., those who were willing or very willing to engage with the vignette protagonist).

**Table 2:** Desire for social distance by vignette version.

	Gambling without harm	Gambling harm (GH) only	GH + LGBTQ	GH + mental health	GH + drug and alcohol use	GH + minority ethnicity	GH + low income
Mean (SD) total desire for social distance score from…	10.4 (4.2)	13.8 (3.5)	13.6 (4.2)	13.4 (3.7)	16.6 (4.1)	13.6 (3.8)	13.7 (3.7)
Willing to move next door to…	89.1%	77.6%	76.5%	76.1%	36.9%	75.2%	75.0%
Willing to make friends with…	89.5%	69.7%	70.6%	70.6%	36.9%	71.5%	67.6%
Willing to spend an evening with…	87.7%	68.9%	69.0%	72.0%	36.7%	70.9%	64.9%
Willing to start working closely on a job with…	83.9%	57.8%	61.5%	63.6%	33.6%	58.4%	57.6%
Willing to have a residential treatment centre in your neighbourhood for…	76.5%	77.2%	76.7%	75.9%	63.7%	77.8%	78.1%
Willing to have… marry into your family	75.5%	31.1%	38.5%	37.8%	17.3%	30.7%	32.9%

Post-hoc tests confirmed that there was a significantly greater desire for social distance in response to all vignettes describing someone experiencing gambling harms compared to the ‘gambling without harms’ condition (all *p*-values <0.001). In addition, the individual described as having drug- and alcohol-related difficulties alongside gambling harms was met with significantly more stigma (in the form of desire for social distance) than *all* other vignettes (all *p* values <0.001). In general, people are most willing to accept a residential treatment centre for people experiencing gambling harms (between 64 and 78% were willing to have this), and least willing to accept a person experiencing gambling harms marrying into the family (between 17 and 39% were prepared to accept this).

### Predictors of desire for social distance from someone experiencing gambling harms

To identify predictors of desire for social distance, hierarchical linear regression was carried out in SPSS (v.29), in two blocks, using the ‘enter’ method, using responses to all vignettes depicting someone experiencing gambling harms, *n* = 3,052. Firstly, to explore how beliefs about gambling harms and those who experience them influence desire for social distance, block 1 included predictor variables relating to beliefs about nature and origin of gambling harms, with desire for social distance from vignette protagonist as the criterion variable. The model was statistically significant ((*F* (11, 3,041) = 89.346), *p* < 0.001) and explained 24% of the variance in desire for social distance (*R*^2^ = 0.242). Table 3 presents summary statistics for the predictor variables, including squared semi-partial correlation coefficients (sr^2^), which give a standardised measure of effect size, indicating the amount of unique variance explained by each predictor (Dudgeon, 2016; Tabachnick and Fidell, 2001). In order of decreasing effect size, desire for social distance was predicted by: belief in bad character as a cause of harm; perceived *ir*recoverability (indicated by negative coefficient for recoverability); perceived disruptiveness; perceived harmfulness to others; *dis*belief in god’s will as a cause of harm (as indicated by negative coefficient for belief in god’s will as a cause); and perceived harmfulness to self. The other variables (belief that harms are caused by chemical imbalance, stressful circumstances, genetics or upbringing; and perceived noticeability) did not significantly predict desire for social distance.

**Table 3:** Summary of hierarchical linear regression predicting desire for social distance scores.

Model	Predictor variable	Unstandardized coefficients		Β	T	*p*-value	95% CI	*sr* ^2^
		B	Std. Error					
Block 1: *F* (11, 3,041) = 89.34, *p* < 0.001, *R*^2^ = 0.244.	Belief in bad character cause	**1.144**	**0.073**	**0.284**	**15.624**	**<0.001**	**1.001, 1.288**	**0.061**
	Belief in chemical imbalance cause	−0.088	0.073	−0.023	−1.207	0.228	−0.232, 0.055	0.000
	Belief in stress cause	0.023	0.090	0.005	0.259	0.795	−0.153, 0.199	0.000
	Belief in genetic cause	0.005	0.075	0.001	0.067	0.947	−0.143, 0.153	0.000
	Belief in divine will cause	**−0.222**	**0.065**	**−0.058**	**−3.407**	**<0.001**	**−0.349, −0.094**	**0.003**
	Belief in upbringing cause	0.030	0.070	0.007	0.426	0.670	−0.108, 0.168	0.000
	Perceived harmfulness to others	**0.519**	**0.076**	**0.128**	**6.799**	**<0.001**	**0.370, 0.669**	**0.011**
	Perceived harmfulness to self	**0.191**	**0.085**	**0.042**	**2.249**	**0.025**	**0.025, 0.358**	**0.001**
	Perceived noticeability	0.083	0.075	0.019	1.115	0.265	−0.063, 0.229	0.000
	Perceived recoverability	**−0.970**	**0.082**	**−0.201**	**−11.810**	**<0.001**	**−1.131, −0.809**	**0.035**
	Perceived disruptiveness	**0.222**	**0.023**	**0.165**	**9.548**	**<0.001**	**0.177, 0.268**	**0.023**
Block 2: *F* (18, 3,034) = 66.54, *p* < 0.001, *R*^2^ = 0.283	Belief in bad character cause	**1.080**	**0.072**	**0.268**	**14.950**	**<0.001**	**0.938, 1.221**	**0.053**
	Belief in chemical imbalance cause	−0.013	0.072	−0.003	−0.186	0.852	−0.154, 0.128	0.000
	Belief in stressful cause	0.011	0.088	0.002	0.129	0.897	−0.162, 0.185	0.000
	Belief in genetic cause	−0.076	0.074	−0.019	−1.024	0.306	−0.221, 0.069	0.000
	Belief in divine will cause	−0.121	0.065	−0.032	−1.871	0.061	−0.249, 0.006	0.001
	Belief in upbringing cause	0.123	0.069	0.030	1.774	0.076	−0.013, 0.258	0.001
	Perceived harmfulness to others	**0.555**	**0.075**	**0.137**	**7.404**	**<0.001**	**0.408, 0.702**	**0.013**
	Perceived harmfulness to self	**0.166**	**0.084**	**0.037**	**1.974**	**0.049**	**0.001, 0.331**	**0.001**
	Perceived noticeability	**0.154**	**0.073**	**0.035**	**2.101**	**0.036**	**0.010, 0.298**	**0.001**
	Perceived recoverability	**−0.882**	**0.081**	**−0.183**	**−10.951**	**<0.001**	**−1.040, −0.724**	**0.028**
	Perceived disruptiveness	**0.237**	**0.023**	**0.175**	**10.326**	**<0.001**	**0.192, 0.281**	**0.025**
	Level of contact score	**−0.100**	**0.021**	**−0.082**	**−4.774**	**<0.001**	**−0.140, −0.059**	**0.005**
	Age	**0.037**	**0.004**	**0.158**	**9.582**	**<0.001**	**0.029, 0.044**	**0.022**
	Scores 1 + on PGSI	**−0.582**	**0.180**	**−0.061**	**−3.233**	**0.001**	**−0.935, −0.229**	**0.002**
	Past year gambler with PGSI of 0	−0.108	0.145	−0.013	−0.747	0.455	−0.392, 0.176	0.000
	Affected other	**0.449**	**0.228**	**0.033**	**1.967**	**0.049**	**0.001, 0.896**	**0.001**
	Minority ethnicity	0.257	0.174	0.024	1.480	0.139	−0.083, 0.598	0.001
	Male gender	**−0.339**	**0.127**	**−0.042**	**−2.672**	**0.008**	**−0.587, −0.090**	**0.002**

Bold font indicates statistically significant predictor (*P*< 0.05).

Block 2: In order to explore how key demographics (age, sex and ethnicity) and personal experience with gambling harms (levels of contact scale score, affected other status, PGSI 1 + score, and past year gambling) influence desire for social distance, after controlling for beliefs about gambling harms, another block was added to the hierarchical multiple linear regression, again using the ‘enter’ method. These variables explained an additional 4.1% of the variance (*R*^2^ = 0.283; ΔR^2^ = 0.041). Significant predictors, in order of decreasing effect size, were: age, level of contact (inverse predictor), PGSI 1 + status (inverse predictor), male sex (inverse predictor), and affected other status. While these predictors were significant, it is important to note that the size of these effects are small. The majority of variables from block one remained significant/non-significant within block 2, but belief in divine will was no longer a significant predictor, and ‘noticeability’ which had not been significant, became significant (*p* = 0.036), though the effect size was very small.

Assumption checking was carried out for the regression as a single simultaneous model (to ensure any issues with multicollinearity across blocks would be identified): Tolerance was >0.67 for all variables, and variance inflation factor (VIF) was <1.5 (i.e., well below 10) for all variables, indicating no issues with multicollinearity. Originally, PGSI score was entered into the regression but due to a heavy skew towards 0 and a large number of outliers (>3.0), categorical variables (of PGSI 1 + and past year gambling status with PGSI of 0) were used to represent gambling experience instead. There were some slight outliers in other predictor variables (−3.7 to +3.2) but inspection of the cases revealed no reason to discount these scores as there was no evidence of errors. Standardised residuals were randomly distributed relative to the predicted values of the DV (as indicated by scatterplot) and normally distributed (as indicated by histogram) and ranged from +3.67 to −3.36 (11 cases over 3, and 4 cases below −3). Again, inspection of the cases revealed no data errors and there was no obvious reason to doubt the validity of these cases, so all values were retained in calculating the statistics reported. While some (123) values had a leverage of more than 2*(number of predictors/sample size), (i.e., >0.0118), and 20 had a value more than 3* the number of predictors/sample size (>0.0177), Cook’s distance was <0.011 for all cases, indicating that it was unlikely that the model was biased by highly influential data points. Furthermore, repeating the analysis without the 123 values with a high leverage value (i.e., >0.0118), the model remained significant with the same *p*-values and a similar R^2^ values, and the same predictors remained significant/non-significant, with the exception of ‘affected other’ status which fell below the *p* < 0.05 threshold, likely due to the reduced statistical power, given the small number of people in this category. Therefore, the results reported are based on the full sample.

## Discussion

In order to better understand the factors predicting stigmatisation of people experiencing gambling harms in a large general population sample in Great Britain, the current survey used a between-subjects design with vignettes depicting a person experiencing gambling harms alone or alongside one of several potentially stigmatised socio-demographic characteristics, along with questions about participants’ own demographic characteristics, their contact with and experience of gambling/gambling harms, and their beliefs about the nature and origin of gambling harms.

As predicted, people expressed, on average, a greater desire for social distance from a hypothetical individual experiencing gambling harms, when compared with someone gambling without harm. This is consistent with the extant literature on stigma in relation to gambling harms, confirming that, while gambling is normalised and accepted as a recreational activity, there is stigmatisation of those who experience harms (Wöhr and Wuketich, 2021). On a positive note, the proportion of people who were willing to engage with someone experiencing gambling harms was notably higher than was seen in a study using similar vignettes and outcome measures, conducted in Australia in 2016 (Hing et al., 2016a). For example, around 70% of our sample were willing to make friends with someone experiencing gambling harms, and while this was lower than the 90% who were willing to make friends with someone gambling without harms, it was considerably higher than the 36% of people in Hing et al.’s study who were willing to do this (despite the vignettes being almost identical). This could be a cultural difference, and/or a result of reduced stigma over the past 8 years; due to a lack of appropriate comparison data from UK samples in recent years it is challenging to determine which of these explanations is most likely. However, monitoring for future fluctuations in desire for social distance from the benchmarks in this study could be useful to identify potential changes in societal attitudes over time, e.g., in response to national stigma reduction campaigns such as those conducted by GambleAware in 2023 and 2024.

In contrast to predictions that providing participants with information about additional (potentially stigmatised) characteristics of the person in the vignette would increase stigmatisation, we did not find evidence of a significant increase in people’s desire for social distance when the person was also described as being from a minority sexuality (LGBTQ) or ethnicity background; from a low income household, or experiencing mental health difficulties. It is possible that this indicates a genuine lack of intersectional stigmatisation of people with these characteristics. This could be due to stigmatisation of gambling harms overshadowing other existing stigmatisation, or due to absence of stigmatisation of these characteristics – though given that predictions of stigmatisation of these characteristics were based on existing empirical literature, the latter explanation seems less likely. It is also possible that socially desirable responding could partially explain this finding, with some participants being conscious that it is socially unacceptable to stigmatise individuals from these groups, and managing their responses accordingly. While somewhat more challenging to administer, implicit measures can be a useful means of evaluating people’s attitudes in instances such as this, where impression management may be obscuring people’s true beliefs (Anderson, 2019), so further research exploring intersectional stigma using such measures would be valuable in exploring this possibility. This may also reveal ‘hidden’ stigmatisation of people experiencing gambling harms, beyond that seen in the desired social distance scores reported in this study.

Social desirability concerns in responding could also partially explain why there was evidence of compounded stigmatisation of the protagonist experiencing gambling harms in conjunction with long-term drug and alcohol use, as stigmatisation and discrimination of people with substance and/or alcohol use-related difficulties is not only prevalent, but has been found to be more socially accepted - compared with disorders unrelated to substance use (Kilian et al., 2021). This finding has implications for stigma reduction efforts, and for informing individuals working in education and support services with people experiencing gambling harms alongside drug and alcohol use of the likelihood of a high burden of stigma in this population.

There were no significant differences in desire for social distance from male compared with female vignette protagonists, which is consistent with other studies that have not found a consistent difference in the perceived stigmatisation of males and females experiencing gambling harms (Andrà et al., 2022). However, it is important to note that this does not negate the possibility of gender differences in individuals’ experiences of stigmatisation. For example, the vignette responses cannot tell us about self-stigma, and some studies have found greater self-stigma amongst females experiencing gambling harms, compared with males (Hing et al., 2015; Hing and Russell, 2017b). Thus, these findings do not negate the importance of prioritising interventions for groups, such as women, that have been identified as being more likely to internalise stigma (Quigley, 2022). The relative lack of contextual information about the protagonist is also important to acknowledge. For example, participants were not provided with any detail about the individual’s age or carer status, and being a mother is one factor thought to drive elevated stigma amongst females experiencing gambling harms (Collinson and Hall, 2021; Estévez et al., 2023; Fannin et al., 2024), thus, there would still be value in further exploration of potential gender differences in stigmatisation of gambling harms.

Merging data across all vignettes describing someone experiencing gambling harms, we identified several significant predictors of desire for social distance; many of which replicated the findings of Hing and colleagues (2016). Specifically, the more disruptive and harmful the person experiencing harms was perceived to be, and the less possible they thought it was for them to recover, the greater the stigmatisation. Participants were also more likely to stigmatise someone experiencing gambling harms if they felt their circumstances were caused by their bad character. All of these findings are consistent with those of Hing et al., and can be useful in informing stigma reduction interventions, as they highlight key drivers of stigma. While Hing and colleagues found that belief in stressful life circumstances or chemical imbalance as a cause of gambling harms predicted lower desire for social distance, these causes did not emerge as being statistically significant in our sample. This may suggest that within the UK, stigma reduction efforts would benefit more from focusing on combating the belief that gambling harms are driven by bad character, than on emphasising specific causes related to stress or chemical imbalance. However, see Quigley for a nuanced discussion of considerations in stigma reduction interventions (Quigley, 2022).

These findings can also be understood within the wider policy and ideological context in the UK, where responsibility for gambling harms has historically been framed in largely individualistic terms. This aligns with a broader neoliberal paradigm that emphasises personal choice, autonomy, and accountability, which may reinforce beliefs that gambling harms are the result of personal failings or bad character. Such framing may obscure the structural and commercial determinants of gambling harm, including the role of the gambling industry, it’s practices and gaps in regulation.

Recent changes in UK gambling policy, which include a shift towards a more public health-oriented approach [as seen in the 2023 Gambling White Paper (Department for Culture, Media and Sport, 2023)], demonstrate increasing recognition of the need to address gambling harms at a systemic level. Public awareness campaigns (such as recent stigma campaigns over the past 3 years, by GambleAware) may also help counteract stigma by reframing gambling harm as a health and social issue rather than a personal moral failing. Monitoring how such changes impact public perceptions and stigmatisation over time would be a valuable direction for future research.

In terms of the characteristics of participants, age was associated with increased desire for social distance, consistent with our predictions and with existing literature (Rockloff and Schofield, 2004; Hing et al., 2016a). Interestingly, male sex was associated with significantly less desire for social distance in our study, despite not emerging as a significant predictor similar vignette studies (Hing et al., 2016a; Brown and Russell, 2020). Given the relatively small size of this effect and the fact it was not predicted *a priori*, it should be treated with caution, but if replicated in future studies, it may bear further investigation, for instance through qualitative research, in order to understand why males may be more willing to engage with someone experiencing gambling harms.

We replicated previous findings of an inverse relationship between level of prior contact with gambling harms and desired social distance (Dhillon et al., 2011), supporting the idea that familiarity can reduce stigmatisation. This is also consistent with the idea that contact interventions can help to reduce stigmatisation, though again see Quigley for a discussion of differential success of such approaches (Quigley, 2022). Affected other status was associated with small but statistically significant *increased* desire for social distance, which demonstrates that prior contact with someone experiencing gambling harms does not universally reduce desired social distance, and is consistent with the idea that burden of gambling harms upon those close to someone experiencing them can create a ‘U’ shaped relationship between familiarity and stigma (Corrigan and Nieweglowski, 2019).

We found that those with prior experience of gambling harms themselves had significantly lower desired social distance from the person in the vignette experiencing harms than those without any experience of harms. While this is broadly consistent with findings that personal experience of gambling predicted lower stigmatisation of gambling harms (Hing et al., 2016b) our prediction of this effect was only tentative, given the possibility that the projection of self-stigma towards others experiencing gambling harms, and/or self-group distancing (van Veelen et al., 2020) could have had an opposite effect. It is important to note that this finding does not negate the possibility of this mechanism operating in some individuals experiencing gambling harm, but overall, in our sample, lived experience of harms predicted reduced desire for social distance. This tendency for higher levels of acceptance amongst peers with similar experiences of harms may be one reason why peer support is reported as being a valuable resource during recovery (Nilsson et al., 2023).

It is important to note that, while the demographic characteristics of the sample were broadly representative of the population of Great Britain, the sample cannot be claimed to be fully representative, due to merging of the ‘main sample’ of YouGov’s panel with their ‘boost’ sample of participants with experiences of gambling. This was a pragmatic decision to maximise the sample size and afford sufficient statistical power to undertake comparisons across several vignette conditions, but replication in a fully representative sample would be beneficial.

This study is prone to the typical limitations of a vignette design, i.e., respondents’ attitudes towards a hypothetical individual may not be a full representation of their attitudes/behaviours towards people within the real world, and, as mentioned already, socially desirable responding may have obscured the degree of stigmatisation. However, strengths include the large sample size, and use of several measures that have been validated in other studies of people experiencing gambling harms (Hing et al., 2016a). Findings have utility for stigma reduction interventions, and can also inform those working with people experiencing gambling harms. In addition to highlighting the ongoing issue of stigmatisation of gambling harms in general, the results have particular relevance for service providers whose roles involve interactions with individuals experiencing gambling harms alongside drug and/or alcohol use. It is important to recognise the likelihood of a high burden of stigma in this population, which may deter people from treatment seeking, and may be an important target during treatment/support provision, given the negative impact that stigma has on wellbeing (Langham et al., 2015). It is also important to evaluate and address potential systemic stigmatisation of this population, given the fact that stigmatisation by service providers can have a negative impact on the quality of care (Aronowitz and Meisel, 2022).

As highlighted by our finding that personal experience of gambling harms inversely predicts stigmatisation of others who experience gambling harms stigma reduction interventions need to target various audiences, rather than simply those experiencing gambling harm, including the broader public, media professionals, healthcare and support service providers, and ‘affected others’. Public awareness campaigns (such as recent campaigns by GambleAware), have the potential to play an important role in reframing gambling harm as a public health issue rather than a personal failing. It is important to ensure that such campaigns use non-stigmatising language and imagery, particularly given the role media representations play in shaping public attitudes (Goffman, 1963).

Within healthcare and support services, such campaigns could include staff training programmes to help reduce implicit biases and promote person-centred, compassionate care. For ‘affected others’, peer support initiatives could be used to help reduce stigma-driven blame or shame, and improve supportive communication. Future work should investigate how these approaches can be adapted and evaluated within the context of gambling harms in the UK.

## Data Availability

The raw data supporting the conclusions of this article will be made available by the authors, without undue reservation.
